# Comparative Analysis between *Salmonella enterica* Isolated from Imported and Chinese Native Chicken Breeds

**DOI:** 10.3390/microorganisms11020390

**Published:** 2023-02-03

**Authors:** Wenjian Shi, Wenli Tang, Yafei Li, Yu Han, Lulu Cui, Shuhong Sun

**Affiliations:** 1 Shandong Key Laboratory of Animal Biotechnology and Disease Control and Prevention, College of Animal Science and Veterinary Medicine, Shandong Agricultural University, Tai’an 271018, China; 2 Shandong Provincial Key Laboratory of Quality Safety Monitoring and Risk Assessment for Animal Products, Shandong Center for Quality Control of Feed and Veterinary Drug, Jinan 250100, China

**Keywords:** *Salmonella enterica*, public health, native breed, imported breed, ESBLs, antimicrobial resistance, resistance gene

## Abstract

*Salmonella enterica* is considered a significant threat to the global poultry industry and public health. In recent decades, antimicrobial resistance in *Salmonella enterica* has attracted increasing concern throughout the world. However, limited information is available on *Salmonella enterica* among different breeds of breeder chickens. Thus, this study aimed to compare the prevalence, serotype distribution, emergence of extended-spectrum beta-lactamases (ESBLs), antimicrobial resistance, and genetic resistance mechanisms in *Salmonella enterica* among different breeds of breeder chickens. A total of 693 samples (dead embryos, cloacal swabs, water, feed, environmental swabs, and meconium of newly hatched chicks) were selected and cultured for *Salmonella* from four breeder chicken farms in Shandong province, China, representing one imported and three native breeds, and the isolates were further serotyped. Of the *Salmonella* isolates, susceptibility to 11 antimicrobials of 5 classes, ESBL screening, and the presence of 21 antimicrobial resistance genes were determined in the present study. Overall, 94 (13.6%) isolates were recovered, which were divided into 3 serotypes (*Salmonella* Pullorum (*n* = 36), *Salmonella* Thompson (*n* = 32), and *Salmonella* Enteritidis (*n* = 26)). The results showed that the prevalence of *Salmonella enterica* isolates from the imported breeds was higher compared with the three domestic breeds. Eight of the ninety-four isolates were ESBL-positive strains, which were recovered from a domestic breed chicken farm. These eight ESBL-producing isolates were serotyped to Pullorum. Surprisingly, *Salmonella* Enteritidis (*S. enteritidis*) and *S. pullorum* were simultaneously isolated from a single dead embryo observed among one native breed. Meanwhile, among the *Salmonella* isolates, 53.2% (50/94) were multidrug-resistant strains, and 44.7% (42/94) of the isolates presented resistance to at least five antibiotics. Nearly all of the isolates (97.9%, 92/94) were resistant to at least one antimicrobial; one isolate of *S*. Thompson was resistant to seven antimicrobial agents belonging to four different classes. The carriage rate of three resistance genes (*tetA*, *tetB*, and *sul1*) among isolates from the imported breeds (87%, 70%, and 65.2%) was higher than that in those from domestic breeds (35.2%, 36.6, and 14.1%). To our knowledge, this is the first report of ESBLs-producing *Salmonella* isolated from a Chinese native breed of breeder chickens. Our results also highlight that a high prevalence of multidrug-resistant *Salmonella enterica* contamination is widespread among different breeds of breeder chickens, which is a major risk of food-borne diseases and public health.

## 1. Introduction

*Salmonella* spp. are recognized as zoonotic pathogens and one of the leading agents capable of infecting humans and animals [1]. Contaminated poultry and poultry products are essential sources of food-borne illnesses in humans [2,3]. There are several serotypes of *Salmonella* spp., and more than 3000 serotypes have been classified according to the response of antisera to O and H antigens [4,5] Among *Salmonella* spp., more than 2500 serovars belong to *Salmonella enterica*, which has become a severe threat to public health and the poultry industry [6]. The World Health Organization (WHO) estimates that, in low- and middle-income countries, about 600 million people fall ill from unclean food each year, causing 420,000 deaths and costing up to USD 110 billion annually [7]. *Salmonella* spp. is also one of the leading causes of significant economic losses in China and around the world [8,9]. The predominant *Salmonella* serotypes prevalent in Chinese native chicken breeds are *Salmonella enterica* serovar Enteritidis and *Salmonella enterica* serovar Gallinarum biovar Pullorum [10]. Among them, *S. pullorum* is widespread and difficult to eliminate because of its vertical transmission [11]. In recent years, there has been some success in implementing rigorous eradication programs for Pullorum, such as attenuated vaccines against host-specific serotypes [12,13]. Many developed countries, such as the United States of America, have been declared free of *S. pullorum*, but it is still widespread in China [14].

The long-term effects of antibiotics may increase resistance to antimicrobials [15,16,17,18]. Quinolones, third-generation cephalosporins, ampicillin, cotrimoxazole, and doxycycline are used for treating *Salmonella* infections. These antimicrobial determinants are horizontally transmitted to other pathogens through a variety of mechanisms [16,19], including chromosomally encoded mechanisms or plasmid-mediated resistance mechanisms [20]. Multidrug-resistant (MDR) bacteria pose a serious threat to public health, with humans or animals at risk of infection when exposed to contaminated poultry or products [21,22,23]. The widespread use of tetracycline in animals since the 1950s has resulted in the development of tetracycline resistance in *Salmonella* spp. [24,25]. Recent studies have shown that *Salmonella* strains isolated from several countries (China, Thailand, the Republic of Bulgari, and Denmark) carry extended-spectrum β-lactamases (ESBLs), which have attracted much attention around the world [26,27,28,29]. ESBL strains complicate antibiotic therapy [30] as ESBL-encoding plasmids can carry additional β-lactamase genes and other antibiotic resistance genes, which may limit treatment options for ESBL-producing pathogens [31]. Both the US Centers for Disease Control and Prevention (CDC) [32] and the WHO [33] list Gram-negative bacteria that produce beta-lactamases as one of the world’s most pressing threats.

Enterobacteria repetitive intergenic consensus polymerase chain reaction (ERIC-PCR) uses consensus primers to amplify DNA sequences located between repeated sequences for subtyping Gram-negative enteric bacteria. Therefore, acknowledging the genetic relationship between different breeds of breeder chickens MDR *Salmonella* isolates using the ERIC-PCR strain is crucial for both humans and animals.

Shandong Province is one of the largest Chinese poultry producers, which exports to several countries. High isolation rates of *Salmonella* recovered from dead embryos and the meconium of newly hatched chicks were confirmed by our previous findings [10,34]. Currently, there are very few studies regarding *Salmonella* in breeder farms’ chickens in Shandong Province, China. Therefore, in order to fill in the epidemiological gaps concerning the distribution of *Salmonella* in different breeder chicken farms, four different breeder farms were chosen for the identification and serotyping of *Salmonella* isolates. Above all, to the best of our knowledge, this is the first report of ESBL-producing *S. pullorum* being isolated from Chinese native chicken breeds.

## 2. Materials and Methods

### 2.1. Sampling Strategy and Isolation and Identification of Salmonella

All animal work was reviewed and approved by the Laboratory Animal Care Committee of Shandong Agricultural University (permit number SDAUA-2021-034) on 10 March 2022. Informed verbal consent was obtained from farmers to allow samples to be collected from their farms.

In July 2020, a total of 693 samples (458 dead embryos, 100 cloacal swabs, 20 water samples, 15 feed samples, 30 environmental swabs, and 70 meconium samples of newly hatched chicks) were selected from four different chicken breeds in Tai’an (White feather broiler), Jinan (Bairi), Rizhao (Langya), and Jining (Luhua) in Shandong Province, China, for *Salmonella* isolation and identification. The isolation and identification of *Salmonella* species were performed according to ISO standard 6579:2017 and the Chinese national standard (GB 4789.4-2016) with minor modifications [35,36]. Each sample was added to 4.5 mL of buffered peptone water (BPW, Qingdao Hope Bio-technology Co., LTD., China) and the BPW mixture was incubated at 37 °C for 12 h for pre-enrichment. Approximately 0.5 mL of pre-enriched culture was inoculated into 4.5 mL of Tetrathionate Broth Base (TTB, Qingdao Hope Bio-Technology Co., Ltd., Qingdao, China) and Selenite Cystine Broth (SC, Qingdao Hope Bio-Technology Co., Ltd., Qingdao, China). Cultures of each TTB and SC broth were inoculated on a Xylose Lysine Desoxycholate Agar base (XLD, Qingdao Hope Bio-technology Co., Ltd., Qingdao, China) and incubated at 37 °C for 48 h [37]. Smooth and round without a black center or large with a black center colonies were confirmed by polymerase chain reaction (PCR) assays with primers designed for the *FimW* gene (Table S1) [10,38]. Bacterial DNA was extracted using a TIANamp Bacterial DNA Kit (TIANGEN, Beijing, China) according to the manufacturer’s instructions.

Amplification reactions were carried out at a final volume of 25 µL containing 1 µL of each primer (10 µM), 12.5 µL of 2 × Taq Master Mix (Vazyme Biotech, Nanjing, China), 1 μL of genomic DNA, and 9.5 μL of distilled deionized water. PCR of the *FimW* gene was performed with the following program: 94 °C for 4 min, followed by 24 cycles of 1 min at 94 °C, 1 min at 50 °C, and 1 min at 72 °C, and a final extension at 72 °C for 10 min. The PCR products were separated through 1% agarose gel electrophoresis and visualized under UV light after staining with ethidium bromide. To confirm the presence of *Salmonella*, the standard strain of *S*. *enterica* (CVCC 3377) purchased from the China Center for the preservation and management of veterinary microorganisms was used as a positive control, and *Escherichia coli* (ATCC 25922) was used as a negative control.

### 2.2. Serological Identification and Biochemical Identification of Salmonella Isolates

All isolates were serotyped by slide agglutination of O and H antigens according to the instructions provided by the manufacturer of the antiserum that we used (Ningbo Tianrun Bio-technology Co., LTD., China). The serotypes could then be determined according to the Kauffmann–White classification scheme [39]. For the identification of *S*. *gallinarum* and *S. pullorum*, dulcitol fermentation and ornithine decarboxylation tests were conducted according to a previous study [40]. *Salmonella* biochemical identification tubes were purchased from Hangzhou Microbial Reagent Co., Ltd. (Hangzhou, China). PCR was performed on isolates identified as *S. pullorum* using a specific *IpaJ* gene for *S. pullorum* (Table S1); the standard strain of *S*. *pullorum* (CVCC 526) purchased from the China Center for the Preservation and Management of Veterinary Microorganisms was used as a positive control and *S. enteritidis* (CVCC 3377) was used as a negative control.

### 2.3. Antimicrobial Susceptibility Testing

According to the Clinical and Laboratory Standards Institute (CLSI) protocol [41], the Kirby–Bauer drug-sensitive disk method was used to test the sensitivity of *Salmonella* isolates to 11 antimicrobials of 5 classes, including β-lactames: ampicillin (AMP), 10 μg; amoxicillin (AMX), 20 μg; ceftazidime (CAZ), 10 μg; and cefoxitin (FOX), 30 μg; tetracyclines: tetracycline (TET), 30 μg and doxycycline (DOX), 30 μg; quinolones: ofloxacin (OF), 5 μg; aminoglycosides: gentamicin (GM), 10 μg and streptomycin (STR), 10 μg; and sulfonamides: sulfamethoxazole–trimethoprim (SXT), 25 μg. Briefly, bacterial suspensions were obtained from overnight cultures, adjusted to the 0.5 McFarland turbidity standard, and the organisms were then evenly spread on the surface of a Muller Hinton agar plate using a cotton swab. After about 15 min, the disks were applied to the plates and incubated at 37 °C for 18 h. Finally, the diameter of the inhibition zone was measured using a ruler. As per standardized international terminology created by the European Centre for Disease Control (ECDC) and CDC, multidrug-resistant (MDR) bacteria were defined as having acquired nonsusceptibility to at least one agent in three or more antimicrobial categories [42].

### 2.4. ESBL Screening

Cefotaxime and ceftazidime were used as the initial screening standards, and strains resistant to both cefotaxime and ceftazidime were selected for ESBL screening. The double-disk synergy method was designed for drug susceptibility testing. ESBLs have the property of being inhibited by clavulanate. Bacteriostatic rings were found in resistant strains around the mixed discs of third-generation cephalosporins and clavulanate. If clavulanate increased the bacteriostatic ring by more than 5 mm, the isolates were presumed to produce ESBLs. *Escherichia coli* (ATCC 25922) and *Klebsiella quasipneumoniae* (ATCC 700603) were used as negative and positive controls, respectively.

### 2.5. Molecular Detection of Antimicrobial Resistance-Associated Genes

All isolates were screened for the presence of 21 antimicrobial resistance genes. The following genes encode resistance to β-lactamases: *bla*_VIM_, *bla*_SHV_, *bla*_NDM_, *bla*_CTX-M_, *bla*_TEM_, *bla*_IMP_, *bla*_MIR_*,* and *bla*_DHA_; aminoglycosides: *AAC2* and *AAC4*; quinolones: *qnrC* and *oqxA*; tetracyclines: *tetA*, *tetB*, and *tetC*; sulfonamides: *sul1* and *sul2*; and colistin: *Mcr-1*, *Mcr-2*, *Mcr-3*, and *Mcr-4*. For each gene, PCR was performed in a final 25 μL reaction mixture system containing 12.5 μL of 2 × Taq Master Mix (Vazyme Biotech co., Ltd., Nanjing, China), 10 μM of forward and reverse primers, 1 μL of genomic DNA, and 9.5 μL of distilled deionized water. The primers used for 21 antimicrobial resistance genes are detailed in Table S2. The PCR conditions and size of the PCR products were obtained as previously described (Table S3) [28,43,44,45,46,47,48,49,50].

### 2.6. ERIC-PCR 

The primers (F: 5′-ATG TAA GCT CCT GGG GAT TCA C-3′, R: 5′-AAG TAA GTG ACT GGG GTG AGC G-3′) were used for ERIC-PCR [51]. The PCR was performed in a 25 μL solution containing 1 μL of each primer (10 μM), 2 μL of genomic DNA, 8.5 μL of distilled deionized water, and 12.5 μL of 2 × Taq Master Mix (Vazyme Biotech, Nanjing, China). The PCR conditions were as follows: 1 cycle at 94 °C for 7 min, followed by 35 cycles of 45 s at 94 °C, 1 min at 52 °C, and 8 min at 65 °C, and a final extension at 65 °C for 10 min. The same reaction mixture without a DNA template was used as the negative control. ERIC-PCR products were electrophoresed on 1.5% agarose gel, and the gel images were analyzed using a Kodak Gel Logic 212 Imaging System (Rochester, NY, USA). The product sizes were estimated using a DNA ladder 5000 (Vazyme Biotech, Nanjing, China).

The ERIC patterns of each isolate were coded as 1 (presence) or 0 (absence) for DNA bands. Dendrograms of each isolate were constructed using the unweighted pair–group method with an arithmetic average (UPGMA). Additionally, isolates were considered to have the same ERIC patterns with more than 80% genetic similarity, and isolates with more than 90% similarity were treated as the same strains.

### 2.7. Statistical Analysis

The statistical software package SPSS 23.0 (SPSS, Inc., Chicago, IL, USA) was used for data analysis. To compare the differences between the results obtained from imported and native breeds, a chi-square test was used.

## 3. Results

### 3.1. Prevalence of Salmonella Isolates

**Figure 1:**
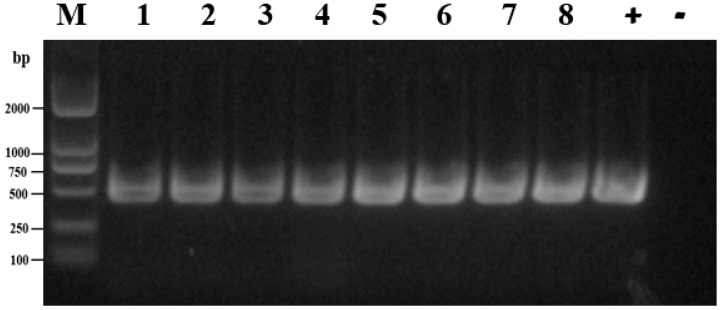
PCR assays for the detection of *Salmonella* spp. isolates. M: molecular weight standards of DL 2000; Lane 1–8: Eight *Salmonella* strains; +: positive control (*S. enteritidis* CVCC 3377); −: Negative control (*Escherichia coli* ATCC 25922).

As is shown in Table 1, a total of 94 *Salmonella* isolates were recovered from 693 samples, in which *Salmonella* was detected from dead embryos, cloacal swabs, feed, and the meconium of newly hatched chick samples. The separation proportion of *Salmonella* isolated from different sample types during the study was from 0 to 38.3%. Among the isolates, *Salmonella* strains were observed in 38.3% (23/60) of those from Farm A, 6.3% (15/238) of those from Farm B, 13.9% (32/230) of those from Farm C (twenty- nine from the dead embryos, one from the cloacal swabs and two from the feed) and 14.5% (24/165) of those from Farm D. The prevalence of *Salmonella* was 38.3% (23/60) from a foreign breed (white feather broiler). Moreover, a high prevalence (34.3, 24/70) of *Salmonella* was also observed in the meconium of newly hatched chicks. The detection results of some samples amplifying the *FimW* gene are shown in Figure 1.

### 3.2. Distribution of Salmonella Serotypes

**Figure 2:**
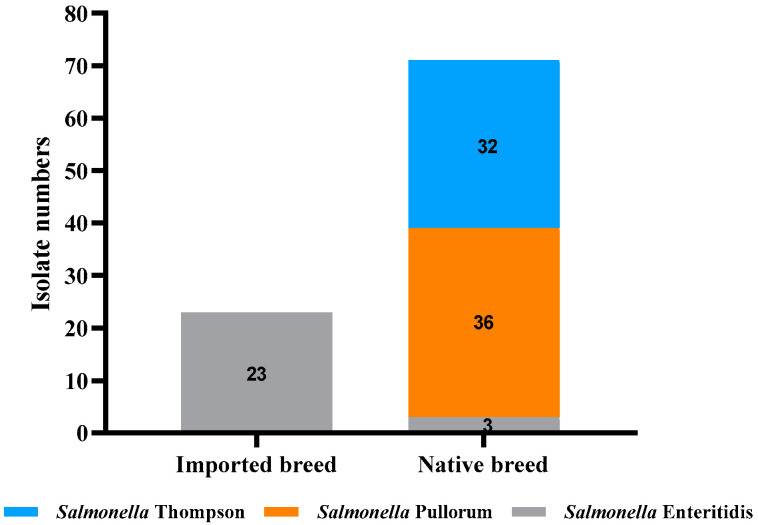
Serotypes of *Salmonella enterica* isolates (*n* = 94) recovered from different chicken breeds.

**Figure 3:**
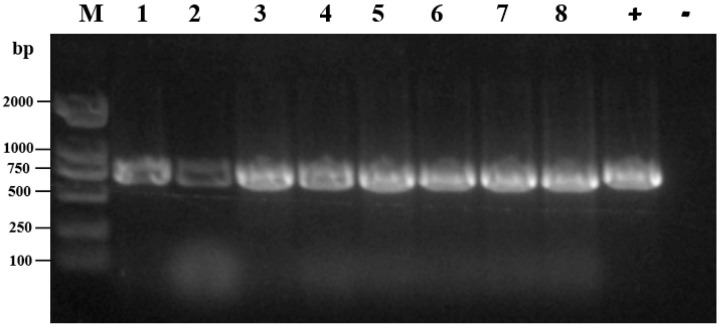
PCR assays for the detection of *Salmonella* spp. isolates. M: molecular weight standards of DL 2000; Lanes 1–8: Eight *Salmonella* strains; +: positive control (*S. pullorum* CVCC 526); −: Negative control (*S. enteritidis* CVCC 3377).

With regard to the serotyping of *Salmonella* isolates, three serotypes of *Salmonella enterica* were identified among the 94 *Salmonella* strains (Figure 2). The isolates from three native breeds of breeder chickens contained three serotypes, and the isolates from foreign breed chicken (white feather broiler) contained only one serotype. The predominant serotypes were *Salmonella enterica* serovar Gallinarum biovar Pullorum (*S. pullorum*) and Gallinarum (*S. gallinarum*) isolated from native breed chickens (36/94, 38.3%), followed by *Salmonella* Thompson (32/94, 34.0%) and *S. enteritidis*, isolated from both domestic and foreign breed farms (26/94, 27.7%). According to the White–Kauffmann–Le Minor scheme, the 36 *Salmonella* strains were identified as *S. pullorum* and *S. gallinarum*. Combined with the results of dulcitol fermentation, the ornithine decarboxylation test, and the PCR assay, these 36 isolates were further confirmed to be *S. pullorum*. In particular, *S. enteritidis* and *S. pullorum* were simultaneously isolated from a single dead chicken embryo in one native breed of breeder chicken. The detection results of some samples amplifying the *Ipaj* gene are shown in Figure 3.

### 3.3. Antimicrobial Susceptibility and ESBL Production

In this study, resistance to at least one antibiotic was observed in 92 isolates (97.9%). The studied isolates had high resistance to streptomycin (83.0%, 78/94), ampicillin (75.5%, 71/94), and amoxicillin (73.4%, 69/94), while all isolates were susceptible to ofloxacin (see Table S4). Meanwhile, the results showed that the resistances (ampicillin, tetracycline, and doxycycline) of *Salmonella enterica* isolated from imported breeds (100%, 82.6%, and 65.2%) were higher than the resistances of *Salmonella enterica* isolated from three domestic breeds (67.6%, 12.7%, and 8.4%) (*p* = 0.012, *p* < 0.01 and *p* < 0.01), respectively (Table 2), while lower resistance was observed against ceftazidime (0 for the imported breed and 21.1% for the native breeds, *p* < 0.01). Among the 94 *Salmonella enterica* isolates, eight (8.5%) were ESBL-producing strains. Notably, all eight ESBL-positive isolates belonged to *S. pullorum*, which was recovered from a native breed of breeder chickens (Table 3). Meanwhile, high resistance rates were detected for ceftazidime (100%) and streptomycin (87.5%). All eight strains were resistant to at least one antimicrobial, while only one (12.5%, 1/8) isolate was MDR. Moreover, the distribution of antimicrobial resistance varied between the confirmed serotypes among these *Salmonella* strains (Table 4). Furthermore, compared with the Pullorum serotype, Thompson and Enteritidis isolates showed quite high resistance (Table 5).

**Figure 4:**
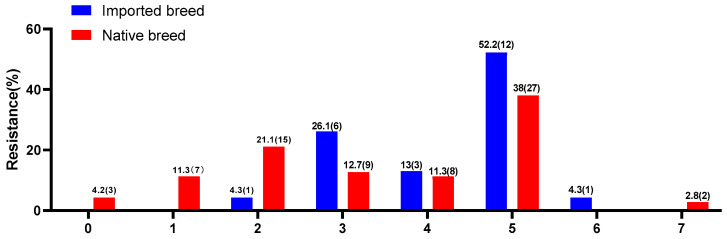
Prevalence of multidrug resistance among 94 *Salmonella enterica* isolates. 0–7: The number of antimicrobials to which the isolates have resistance; (): The number of isolates with resistance to antimicrobials.

The MDR statistics showed that 53.2% (50/94) of strains had multidrug resistance to three or more drug classes. The MDR rates of *Salmonella enterica* isolated from imported breeds (73.9%, 17/23) were much higher than those collected from three native breeds (46.5%, 33/71) (Figure 4). Nearly 85.9% (61/71) of isolates among the three native breeds were resistant to at least two antibiotics, while the resistance rate of *Salmonella* strains in the imported breed was 100% (23/23). Additionally, by comparing the rates of isolates from three domestic chicken breeds (38.0%, *n* = 27) that were resistant to five antibiotics, our results showed that the rates for the strains of *Salmonella* from foreign breeds (52.2%, *n* = 12)) were much higher. On the contrary, two isolates collected from native breeds were resistant to seven antimicrobial agents.

### 3.4. Characterization of Antimicrobial Resistance Genes

The carriage status of *Salmonella* strains for 21 antimicrobial resistance genes was screened by PCR (Table 6). Among these, only one (*bla*_TEM_) of eight genes encoding resistance to β-lactams was detected in 97.9% (92/94) of the isolates. The frequency of the *bla*_TEM_ gene among *Salmonella enterica* recovered in foreign breed farms (100%) was higher than those in native breeds (97.2%) (*p* = 0.841). Among the 85 β-lactam-resistant isolates, all strains harbored the *bla*_TEM_ gene. However, the *AAC2* gene encoding resistance to aminoglycosides was only detected in one isolate collected from an imported breed (*p* = 0.038). With regard to tetracyclines, two (*tetA* and *tetB*) genes were detected in the 28 tetracycline-resistant strains. The rates of three genes (*tetA*, *tetB*, and *sul1*) harbored among isolates from imported breeds of breeder chickens (87%, 70%, and 65.2%) were higher than those of isolates from three domestic breeds (35.2%, 36.6, and 14.1%) (*p* < 0.01, *p* = 0.001, and *p* < 0.01). Furthermore, all instances of two (*sul1* and *sul2*) genes encoding resistance to sulfonamides were detected in the 29 sulfonamide-resistant isolates. Additionally, different serotype isolates carried different resistance genes. Compared with the Enteritidis serotype, Thompson and Pullorum isolates showed higher resistance to *tetA* and *tetB* (Table 7). In addition, no gene encoding resistance to colistin was detected in any of the *Salmonella* isolates.

### 3.5. ERIC-PCR Analysis

**Figure 5:**
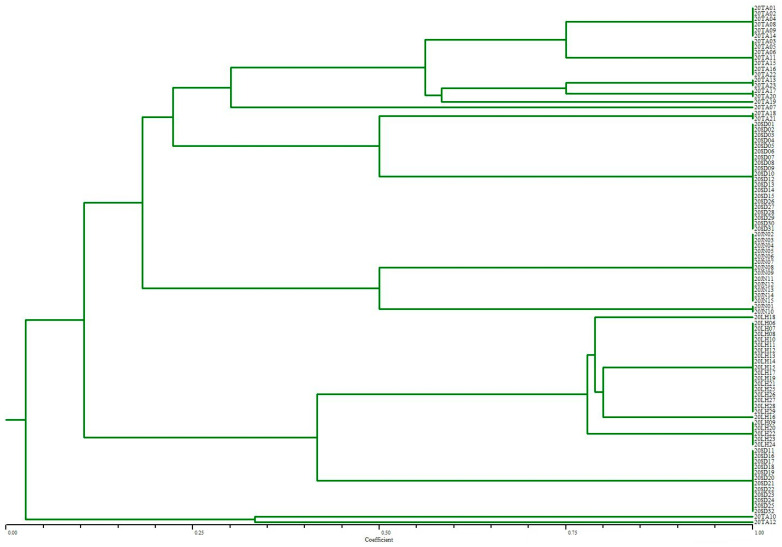
Genetic relatedness of 94 *Salmonella* isolates based on ERIC-PCR patterns. ERIC-PCR dendrogram exhibiting the genetic relatedness of *Salmonella enterica* isolated from four different breeds of breeder chickens. The clustering was performed using UPGMA.

In the present study, the ERIC-PCR results showed that 94 isolates were genotyped into 17 different banding patterns (Figure 5), and isolates belonging to the same serotype were grouped together. The 20 isolates with the serotype Thompson showed a higher correlation, whereas Pullorum and Enteritidis isolates showed genetic diversity. Among the isolates, strains from the same farm were relatively closely clustered. A maximum of 20 Thompson strains belonging to one pattern could be observed. Moreover, 23 isolates recovered from imported breeding chickens were divided into 5 clusters and 2 single isolates, while 71 *Salmonella* strains were classified into 10 clusters, which were recovered from three local breed chicken farms.

## 4. Discussion

*Salmonella* is recognized as one of the most important zoonotic agents in the world, which can be transmitted horizontally and through fertilized eggs [52,53]. Based on this, in order to better evaluate the actual contamination by *Salmonella enterica*, we obtained various samples (dead embryos, cloacal swabs, environment swabs, water, animal feed, and meconium of newly hatched chicks) from four different breeds of breeder chickens.

In this study, the prevalence of *Salmonella enterica* from one imported breed flock and three native breed poultry farms in Shandong Province was 13.6% (94/693); the isolation rate was similar to that in a study on chickens (14.3%) [54], and higher than that from breeder chicken hatcheries in Shandong Province (6.7%) [53]. Additionally, the prevalence of *Salmonella* in the foreign breed (38.6%) was much higher than that collected in Henan Province (11.48%) [52]. In fact, the differences in sample types, geographical locations, sampling years, or isolation methods resulted in a complex and varied isolation comparison.

Three serotypes of *Salmonella enterica* were identified among the 94 *Salmonella* strains according to the Kauffmann–White scheme, and the distribution of serotypes among each breeder chicken was significantly different (Figure 1). *Salmonella* Gallinarum biovar Pullorum collected from two of the three native breed flocks was the most common serotype (38.3%), followed by Thompson (34%) and Enteritidis (27.6%). This finding is consistent with our previous reports [14,55]. These results indicate that, although Pullorum is well controlled in many developed countries, it remains commonly present in Chinese native breeder chickens. *S. pullorum* is ubiquitous in our country and is one of the most serious threats to our poultry industry [56]. Importantly, the prevalence of Pullorum has exceeded 30% in Chinese native breeds of breeder chickens [57] for a long time, resulting in prevention and control work facing great difficulties. *S. enteritidis* is considered one of the most commonly reported pathogens responsible for *Salmonella* infections in humans [58]. Thus, it is important to continuously monitor the prevalence and serovar distribution of *Salmonella* in our domestic breeder chicken farms.

In recent decades, molecular subtyping techniques have been used to widely investigate genetic diversity. Genetic relatedness among *Salmonella* isolates from four different breeds of breeder chicken was analyzed using ERIC-PCR, and the 94 isolates were classified into 17 clusters in this study. In our study, a high level of genetic diversity was observed among the 94 isolates, indicating that there was no significant genetic correlation between the four different breeder chicken farms.

In the current study, most isolates showed high resistance to streptomycin (83.0%, 78/94), ampicillin (75.5%, 71/94), and amoxicillin (73.4%, 69/94), which was similar to some previous studies [52,59,60], suggesting that high selective pressure by exposure to regular use of these antibiotics may be one of the main reasons for the emergence of such antimicrobial-resistant *Salmonella* strains [61]. Interestingly, isolates collected from different breeds of chickens showed different levels of antimicrobial resistance. The overall average antimicrobial resistance of *Salmonella enterica* isolated from three domestic breeds was higher than that of imported breeder flocks, while our study revealed that *Salmonella*, whether from imported breed or native breed chickens, presented low resistance to quinolones regarding the principal antimicrobial agents used in salmonellosis. In addition, 53.2% (50/94) of the isolates were MDR; this result indicated a higher resistance frequency than that described in another investigation [62], which may limit the therapeutic options for *Salmonella* infection. Meanwhile, Enteritidis (100%, 26/26) and Thompson (87.5%, 28/32) showed a high MDR rate in our study; this result was similar to those of other studies [23,60,63]. It is likely to spread along the food production chain with other Enterobacteriaceae through horizontal gene transfer, posing a threat to public health. Of note, the presence of multidrug-resistant isolates may pose a potential transmission risk to public health through the food chain. Antibiotics have been commonly used to prevent and treat diseases in humans and livestock in the past. However, the indiscriminate use of antibiotics in farming, relying on selective pressure, will lead to increased resistance in *Salmonella* and other bacteria, and MDR bacteria might present a significant challenge to the whole poultry industry.

Recent studies showed that the prevalence of ESBL-producing bacteria is increasing [64,65]. In the present study, ESBL positivity was detected in 8.5% (8/94) of the *Salmonella* strains from one native breed in a breeder farm. However, none of them were multidrug-resistant. To our knowledge, this is the first time that ESBL-producing *S. pullorum* has been isolated from a native breed in a breeder chicken farm (Luhua). According to our investigation, the contaminated chickens, poor breeding conditions, and irregular breeding techniques may explain the production of ESBL.

In addition, we found that most of the isolates in this study (97.9%, 92/94) carried the *bla*_TEM_ gene, which is consistent with the high rate of β-lactam resistance. Among the 85 β-lactam-resistant isolates, all strains harbored the *bla*_TEM_ gene, which is regarded as one of the reasons responsible for β-lactam resistance. Our results showed that tetracycline and sulfonamide resistance genes were commonly distributed in most *Salmonella* isolates from different breeds, and this was also correlated with their resistance phenotypes. Tetracycline was discovered in the 1950s and is widely used as a feed additive because of its antibacterial and growth-promoting properties [66]. Additionally, among the tetracycline-resistant strains, the *tetA* and *tetB* genes were detected in all 28 resistant strains; this may be closely related to the abuse of tetracycline. Our study also detected *sul1* and *sul2* genes encoding resistance to sulfonamides in sulfamethoxazole–trimethoprim-resistant isolates. Specifically, tetracycline and sulfonamide AMR genes were detected, but no phenotypic resistance was observed among several strains. Mismatches in genotype and phenotype AMR were also observed in other studies [67,68,69,70]. Indeed, antimicrobial phenotype resistance was not related only to the presence or absence of antimicrobial resistance genes. Several other mechanisms, such as enzyme activation, target modification/protection, and resistance genes, may be considered “silent”; the regulation of resistant gene expression or even a change in the cell wall may affect the phenotype resistance [71]. In contrast, nearly 85.1% of isolates presented a high resistance to aminoglycosides, while only one of them carried the *AAC2* gene encoding resistance to aminoglycosides. Colistin is considered to be the last-resort antibiotic for the treatment of MDR Gram-negative bacteria [72]; none of the four genes encoding colistin resistance were detected in any of the strains.

## 5. Conclusions

In conclusion, multidrug-resistant (MDR) *Salmonella enterica* strains were highly prevalent among different breeds of breeder chickens in Shandong Province, China. This is the first report in which ESBL-producing *S. pullorum* was isolated from a native breed in a breeder chicken farm. In addition, our data found that multidrug-resistant *S. pullorum* and *S.* Thompson are widespread in domestic chicken breeding farms, posing a serious threat to public health. These results highlight the importance of the continuous monitoring of antimicrobial resistance in *Salmonella enterica* among breeder chicken farms in China.

## Figures and Tables

**Table 1:** Number of samples and prevalence of *Salmonella* in Poultry farms (*n* = 693).

Location	Farm	Breed	Type of Sample	No. of Sample	No. of Positive Sample (%)
Tai’an	Farm A	White feather broiler	Dead embryos	60	23 (38.3)
Jining	Farm B	Bairi	Dead embryos	238	15 (6.3)
Rizhao	Farm C	Langya	Dead embryos	100	29 (29)
			Cloacal swabs	100	1 (1)
			Water	15	-
			Feed	15	2 (13.3)
Jining	Farm D	Luhua	Dead embryos	60	-
			Meconium of newly hatched chicks	70	24 (34.3)
			Environmental swabs	30	-
			Water	5	-
			Grand total	693	94 (13.6)

**Table 2:** Numbers and percentages of *Salmonella enterica* isolates resistant to tested antibiotics.

Antibiotic Classes	Antibiotic	Number (%) of Resistant Isolates (*n* = 94)		*p* Value
		Imported Breed	Native Breed	
		(*n* = 23)	(*n* = 71)	
β-lactames	Ampicillin	23 (100)	48 (67.6)	0.012 *
	Amoxicillin	20 (87)	49 (69)	0.150
	Cefoxitin	0 (4.3)	3 (4.2)	0.040 *
	Ceftazidime	0	15 (21.1)	0.000 **
Tetracyclines	Tetracycline	19 (82.6)	9 (12.7)	0.000 **
	Doxycycline	15 (65.2)	6 (8.4)	0.000 **
Quinolones	Ofloxacin	0	0	-
Aminoglycosides	Gentamicin	1 (4.3)	29 (40.8)	0.000 **
	Streptomycin	19 (82.6)	59 (83.1)	0.964
Sulphonamides	Sulfamethoxazole-trimethoprim	0	29 (40.8)	0.000 **

*p* value: * *p* < 0.05; ** *p* < 0.01.

**Table 3:** Characterization of ESBL-producing isolates.

Number	Source	Breed	Serotype	Resistant Phenotype	Resistant Genes
1	Meconium of newly hatched chicks	Luhua	*S. pullorum*	STR—CAZ	*bla*_TEM_—*tetB*
2	Meconium of newly hatched chicks	Luhua	*S. pullorum*	STR—CAZ	*bla*_TEM_
3	Meconium of newly hatched chicks	Luhua	*S. pullorum*	STR—CAZ	*bla*_TEM_—*tetA*—*tetB*
4	Meconium of newly hatched chicks	Luhua	*S. pullorum*	TET—STR—AMX—AMP—CAZ	*bla*_TEM_—*tetB*
5	Meconium of newly hatched chicks	Luhua	*S. pullorum*	STR—AMX—AMP—CAZ	*bla*_TEM_—*tetB*
6	Meconium of newly hatched chicks	Luhua	*S. pullorum*	STR—CAZ	*bla*_TEM_—*tetA*—*tetB*—*sul1*—*sul2*
7	Meconium of newly hatched chicks	Luhua	*S. pullorum*	STR—CAZ	*bla*_TEM_—*tetA*—*tetB*
8	Meconium of newly hatched chicks	Luhua	*S. pullorum*	CAZ	*bla*_TEM_

**Table 4:** Resistance rates of different serovar *Salmonella* isolates toward 10 antimicrobial agents.

Serotypes	Antibiotics									
	SXT	GM	STR	OF	AMP	AMX	DOX	TET	CAZ	FOX
Pullorum	0	0	75%	0	42%	39%	3%	11%	53%	6%
Thompson	91%	91%	91%	0	94%	100%	6%	6%	0	0
Enteritidis	0	4%	85%	0	100%	88%	69%	85%	0%	4%

SXT: sulfamethoxazole–trimethoprim; GM: gentamicin; STR: streptomycin; OF: ofloxacin; AMP: ampicillin; AMX: amoxicillin; DOX: doxycycline; TET: tetracycline; CAZ: ceftazidime; FOX: cefoxitin.

**Table 5:** Rates of multidrug resistance among three different serotypes (%).

Serotypes	Antibiotic Agents							
	0	1	2	3	4	5	6	7
Pullorum	100	100	94	40	27	5	0	0
Thompson	0	0	0	20	46	56	0	100
Enteritidis	0	0	6	40	27	39	100	0

0–7: The number of antimicrobials to which the serotype has resistance.

**Table 6:** Prevalence of antimicrobial resistance genes among *Salmonella* isolates.

Antimicrobial Resistance Genes	Number (%) of Resistant Isolates (*n* = 94)		*p* Value
	Imported Breed	Native Breed	
	(*n* = 23)	(*n* = 71)	
*bla*_TEM_	23 (100)	69 (97.2)	0.841
*AAC2*	1 (4.3)	0	0.038 *
*tetA*	20 (87)	25 (35.2)	0.000 **
*tetB*	16 (70)	26 (36.6)	0.001 **
*sul1*	15 (65.2)	10 (14.1)	0.000 **
*sul2*	0	2 (2.8)	0.094

*p* value: * *p* < 0.05; ** *p* < 0.01.

**Table 7:** Detection rates of antimicrobial resistance genes among the three different serotypes (%).

Serotypes	Antimicrobial Resistance Genes					
	*bla*_TEM_	*AAC2*	*tetA*	*tetB*	*sul1*	*sul2*
Pullorum	100	0	61.1	61.1	22.2	2.8
Thompson	100	3.8	84.6	69.2	65.4	0
Enteritidis	93.8	0	12.5	6.2	0	3.1

## Data Availability

Not applicable.

## Supplementary Materials

The following supporting information can be downloaded at: https://www.mdpi.com/article/10.3390/microorganisms11020390/s1, Table S1: Primers used for confirming *Salmonella* and *S. pullorum*. Table S2: Primer sequences of antimicrobial resistance genes. Table S3: PCR conditions and size of PCR products among 21 antimicrobial resistance genes. Table S4: characteristics of 94 *Salmonella* isolates.

## References

[1] Abd El-Ghany W.A. (2020). Salmonellosis: A Food Borne Zoonotic and Public Health Disease in Egypt. J. Infect. Dev. Ctries.

[2] Ha J.S., Seo K.W., Kim Y.B., Kang M.S., Song C.S., Lee Y.J. (2018). Prevalence and Characterization of *Salmonella* in Two Integrated Broiler Operations in Korea. Ir. Vet. J..

[3] Ammar A.M., Mohamed A.A., Abd El-Hamid M.I., El-Azzouny M.M. (2016). Virulence Genotypes of Clinical *Salmonella*Serovars from Broilers in Egypt. J. Infect. Dev. Ctries.

[4] El-Sharkawy H., Tahoun A., El-Gohary A.E.A., El-Abasy M., El-Khayat F., Gillespie T., Kitade Y., Hafez H.M., Neubauer H., El-Adawy H. (2017). Epidemiological, Molecular Mharacterization and Antibiotic Resistance of *Salmonella enterica* Serovars Isolated from Chicken Farms in Egypt. Gut. Pathog..

[5] Merwad A.M.A., Abdel-Haliem M.E.F. (2018). Isolation and Initial Characterization of a Myoviridae Phage for Controlling Zoonotic *Salmonella* Typhimurium and Salmonella Enteritidis from Broilers in Egypt. Vet. Res..

[6] Paudyal N., Yue M. (2019). Antimicrobial Resistance in the “Dark Matter”. Clin. Infect. Dis..

[7] World Health Organization (WHO) Food Safety. https://www.who.int/en/news-room/fact-sheets/detail/food-safety.

[8] Pan H., Zhou X., Chai W., Paudyal N., Li S., Zhou X., Zhou K., Wu Q., Wu B., Li G. (2019). Diversified Sources for Human Infections by *Salmonella enterica* Serovar Newport. Transbound. Emerg. Dis..

[9] Li Y., Kang X., Ed-Dra A., Zhou X., Jia C., Müller A., Liu Y., Kehrenberg C., Yue M. (2022). Genome-Based Assessment of Antimicrobial Resistance and Virulence Potential of Isolates of Non-Pullorum/Gallinarum *Salmonella* Serovars Recovered from Dead Poultry in China. Microbiol. Spectr..

[10] Song Y., Wang F., Liu Y., Song Y., Zhang L., Zhang F., Gu X., Sun S. (2020). Occurrence and Characterization of *Salmonella* Isolated from Chicken Breeder Flocks in Nine Chinese Provinces. Front. Vet. Sci..

[11] Berchieri A., Murphy C.K., Marston K., Barrow P.A. (2001). Observations on the Persistence and Vertical Transmission of *Salmonella Enterica* Serovars Pullorum and Gallinarum in Chickens: Effect of Bacterial and Host Genetic Background. Avian. Pathol..

[12] OIE Pullorum Disease Timelines (1996–2020). World Organisation for Animal Health (OIE), Paris, France 2020. https://www.oie.int/wahis_2/public/wahid.php/Diseaseinformation/Diseasetimelines.

[13] Zhang-Barber L., Turner A.K., Barrow P.A. (1999). Vaccination for Control of *Salmonella* in Poultry. Vaccine.

[14] Yang X., Huang J., Zhang Y., Liu S., Chen L., Xiao C., Zeng H., Wei X., Gu Q., Li Y. (2020). Prevalence, Abundance, Serovars and Antimicrobial Resistance of *Salmonella* Isolated from Retail Raw Poultry Meat in China. Sci. Total Environ..

[15] Fall-Niang N.K., Sambe-Ba B., Seck A., Deme S.N., Wane A.A., Bercion R., Alambedji-Bada R., Gassama-Sow A. (2019). Antimicrobial Resistance Profile of *Salmonella* Isolates in Chicken Carcasses in Dakar, Senegal. Foodborne Pathog. Dis..

[16] Christaki E., Marcou M., Tofarides A. (2020). Antimicrobial Resistance in Bacteria: Mechanisms, Evolution, and Persistence. J. Mol. Evol..

[17] Varela M.F., Stephen J., Lekshmi M., Ojha M., Wenzel N., Sanford L., Hernandez A., Parvathi A., Kumar S.H. (2021). Bacterial Resistance to Antimicrobial Agents. Antibiotics.

[18] Landers T.F., Cohen B., Wittum T.E., Larson E.L. (2012). A Review of Antibiotic Use in Food Animals: Perspective, Policy, and Potential. Public Health Rep..

[19] Alekshun M.N., Levy S.B. (2007). Molecular Mechanisms of Antibacterial Multidrug Resistance. Cell.

[20] Crump J.A., Sjölund-Karlsson M., Gordon M.A., Parry C.M. (2015). Epidemiology, Clinical Presentation, Laboratory Diagnosis, Antimicrobial Resistance, and Antimicrobial Management of Invasive *Salmonella* Infections. Clin. Microbiol. Rev..

[21] McDermott P.F., Zhao S., Tate H. (2018). Antimicrobial Resistance in Nontyphoidal *Salmonella*. Microbiol. Spectr..

[22] Jajere S.M. (2019). A Review of *Salmonella Enterica* with Particular Focus on the Pathogenicity and Virulence Factors, Host Specificity and Antimicrobial Resistance Including Multidrug Resistance. Vet. World.

[23] Rincón-Gamboa S.M., Poutou-Piñales R.A., Carrascal-Camacho A.K. (2021). Antimicrobial Resistance of Non-Typhoid *Salmonella* in Meat and Meat Products. Foods.

[24] Sheykhsaran E., Baghi H.B., Soroush M.H., Ghotaslou R. (2019). An Overview of Tetracyclines and Related Resistance Mechanisms. Rev. Res. Med. Microbiol..

[25] Lees P., Pelligand L., Giraud E., Toutain P.L. (2021). A History of Antimicrobial Drugs in Animals: Evolution and Revolution. J. Vet. Pharmacol. Ther..

[26] Xiang R., Ye X., Tuo H., Zhang X., Zhang A., Lei C., Yang Y., Wang H. (2018). Co-Occurrence of *Mcr-3* and *Bla(Ndm-5)* Genes in Multidrug-Resistant *Klebsiella Pneumoniae* St709 from a Commercial Chicken Farm in China. Int. J. Antimicrob. Agents.

[27] Liu C., Liu Y., Feng C., Wang P., Yu L., Liu D., Sun S., Wang F. (2021). Distribution Characteristics and Potential Risks of Heavy Metals and Antimicrobial Resistant *Escherichia coli* in Dairy Farm Wastewater in Tai’an, China. Chemosphere.

[28] Song Y., Yu L., Zhang Y., Dai Y., Wang P., Feng C., Liu M., Sun S., Xie Z., Wang F. (2020). Prevalence and Characteristics of Multidrug-Resistant *Mcr-1*-Positive *Escherichia coli* Isolates from Broiler Chickens in Tai’an, China. Poult. Sci..

[29] Archambault M., Petrov P., Hendriksen R.S., Asseva G., Bangtrakulnonth A., Hasman H., Aarestrup F.M. (2006). Molecular Characterization and Occurrence of Extended-Spectrum Beta-Lactamase Resistance Genes among *Salmonella enterica* Serovar Corvallis from Thailand, Bulgaria, and Denmark. Microb. Drug. Resist..

[30] Dinh A., Duran C., Singh S., Tesmoingt C., Bouabdallah L., Hamon A., Antignac M., Ourghanlian C., Loustalot M.C., Pain J.B. (2022). Real-life Temocillin Use in Greater Paris Area, Effectiveness and Risk Factors for Failure in Infections Caused by ESBL-Producing Enterobacterales: A Multicentre Retrospective Study. JAC-Antimicrob. Resist..

[31] Orabi A., Armanious W., Radwan I.A., Girh Z.M.S.A., Hammad E., Diab M.S., Elbestawy A.R. (2022). Genetic Correlation of Virulent *Salmonella* Serovars (Extended Spectrum β-Lactamases) Isolated from Broiler Chickens and Human: A Public Health Concern. Pathogens.

[32] The Centers for Disease Control and Prevention (2013). Antibiotic Resistance Threats in the United States.

[33] WHO (2017). Prioritization of Pathogens to Guide Discovery, Research and Development of New Antibiotics for Drug-Resistant Bacterial Infections, Including Tuberculosis. www.who.int/medicines/areas/rational_use/PPLreport_2017_09_19.pdf?ua=1.

[34] Yang J., Gao S., Chang Y., Su M., Xie Y., Sun S. (2019). Occurrence and Characterization of *Salmonella* Isolated from Large-Scale Breeder Farms in Shandong Province, China. BioMed Res. Int..

[35] (2017). Microbiology of the Food Chain—Horizontal Method for the Detection, Enumeration and Serotyping of Salmonella.

[36] (2016). National Food Safety Standard Food Microbiological Examination: Salmonella.

[37] Zhang F., Song Y., Xu Y., Ju Z., Cui Z., Sun S. (2019). Establishment of Identification Medium and Pcr Assay for *Salmonella* from Poultry. Shangdong J. Anim. Sci. Vet. Med..

[38] Yang B., Niu Q., Yang Y., Dai P., Yuan T., Xu S., Pan X., Yang Y., Zhu G. (2019). Self-made *Salmonella* Pullorum Agglutination Antigen Development and Its Potential Practical Application. Poult. Sci..

[39] Grimont P.A., Weill F.X. (2007). Antigenic Formulae of the *Salmonella* Serovars. WHO Collab. Cent. Ref. Res. Salmonella.

[40] Xiong D., Song L., Pan Z., Jiao X. (2018). Identification and Discrimination of *Salmonella Enterica* Serovar Gallinarum Biovars Pullorum and Gallinarum Based on a One-Step Multiplex PCR Assay. Front. Microbiol..

[41] Clinical and Laboratory Standards Institute (2022). M100, 32nd Ed: Performance Standards for Antimicrobial Susceptibility Testing.

[42] Magiorakos A.P., Srinivasan A., Carey R.B., Carmeli Y., Falagas M.E., Giske C.G., Harbarth S., Hindler J.F., Kahlmeter G., Olsson-Liljequist B. (2012). Multidrug-Resistant, Extensively Drug-Resistant and Pandrug-Resistant Bacteria: An International Expert Proposal for Interim Standard Definitions for Acquired Resistance. Clin. Microbiol. Infect..

[43] Ahmed A.M., Motoi Y., Sato M., Maruyama A., Watanabe H., Fukumoto Y., Shimamoto T. (2007). Zoo Animals as Reservoirs of Gram-Negative Bacteria Harboring Integrons and Antimicrobial Resistance Genes. Appl. Environ. Microbiol..

[44] Poirel L., Walsh T.R., Cuvillier V., Nordmann P. (2011). Multiplex Pcr for Detection of Acquired Carbapenemase Genes. Diagn. Microbiol. Infect. Dis..

[45] Pérez-Pérez F.J., Hanson N.D. (2002). Detection of Plasmid-Mediated Ampc Beta-Lactamase Genes in Clinical Isolates by Using Multiplex Pcr. J. Clin. Microbiol..

[46] Wang M., Guo Q., Xu X., Wang X., Ye X., Wu S., Hooper D.C., Wang M. (2009). New Plasmid-mediated Quinolone Resistance Gene, *QnrC*, Found in a Clinical Isolate of Proteus Mirabilis. Antimicrob. Agents Chemother..

[47] Hansen L.H., Sørensen S.J., Jørgensen H.S., Jensen L.B. (2005). The prevalence of the OqxAB Multidrug Efflux Pump Amongst Olaquindox-Resistant *Escherichia Coli* in Pigs. Microb. Drug Resist..

[48] Sáenz Y., Briñas L., Domínguez E., Ruiz J., Zarazaga M., Vila J., Torres C. (2004). Mechanisms of Resistance in Multiple-Antibiotic-Resistant *Escherichia Coli* Strains of Human, Animal, and Food Origins. Antimicrob. Agents Chemother..

[49] Zhang A.Y., Wang H.N., Tian G.B., Zhang Y., Yang X., Xia Q.Q., Tang J.N., Zou L.K. (2009). Phenotypic and Genotypic Characterisation of Antimicrobial Resistance in Faecal Bacteria from 30 Giant Pandas. Int. J. Antimicrob. Agents.

[50] Carattoli A., Villa L., Feudi C., Curcio L., Orsini S., Luppi A., Pezzotti G., Magistrali C.F. (2017). Novel Plasmid-Mediated Colistin Resistance *Mcr-4* Gene in *Salmonella* and *Escherichia Coli*, Italy 2013, Spain and Belgium, 2015 to 2016. Eurosurveillance.

[51] Wang H., Shu R., Zhao Y., Zhang Q., Xu X., Zhou G. (2014). Analysis of ERIC-PCR Genomic Polymorphism of *Salmonella* Isolates from Chicken Slaughter Line. Eur. Food Res. Technol..

[52] Xu Y., Zhou X., Jiang Z., Qi Y., Ed-Dra A., Yue M. (2020). Epidemiological Investigation and Antimicrobial Resistance Profiles of *Salmonella* Isolated from Breeder Chicken Hatcheries in Henan, China. Front. Cell Infect Microbiol..

[53] Zhao X., Ju Z., Wang G., Yang J., Wang F., Tang H., Zhao X., Sun S. (2021). Prevalence and Antimicrobial Resistance of *Salmonella* Isolated from Dead-in-Shell Chicken Embryos in Shandong, China. Front. Vet. Sci..

[54] Abdeen E., Elmonir W., Suelam I.I.A., Mousa W.S. (2018). Antibiogram and Genetic Diversity of *Salmonella enterica* with Zoonotic Potential Isolated from Morbid Native Chickens and Pigeons in Egypt. J. Appl. Microbiol..

[55] Cui L., Liu Q., Jiang Z., Song Y., Yi S., Qiu J., Hao G., Sun S. (2021). Characteristics of *Salmonella* from Chinese Native Chicken Breeds Fed on Conventional or Antibiotic-Free Diets. Front. Vet. Sci..

[56] Gong J., Zhang J., Xu M., Zhu C., Yu Y., Liu X., Kelly P., Xu B., Wang C. (2014). Prevalence and Fimbrial Genotype Distribution of Poultry *Salmonella* Isolates in China (2006 to 2012). Appl. Environ. Microbiol..

[57] Li H.F., Hu Y., Hu H., Song C., Shu J.T., Zhu C.H., Zhang S.J., Fan J.H., Chen W.W. (2013). Genetic Differ in *Tlr4* Gene Polymorphisms and Expression Involved in *Salmonella* Natural and Artificial Infection Respectively in Chinese Native Chicken Breeds. Mol. Biol. Rep..

[58] Lei C.W., Zhang Y., Kang Z.Z., Kong L.H., Tang Y.Z., Zhang A.Y., Yang X., Wang H.N. (2020). Vertical Transmission of *Salmonella* Enteritidis with Heterogeneous Antimicrobial Resistance from Breeding Chickens to Commercial Chickens in China. Vet. Microbiol..

[59] Zhao X., Yang J., Zhang B., Sun S., Chang W. (2017). Characterization of Integrons and Resistance Genes in *Salmonella* Isolates from Farm Animals in Shandong Province, China. Front. Microbiol..

[60] Elbediwi M., Tang Y., Shi D., Ramadan H., Xu Y., Xu S., Li Y., Yue M. (2021). Genomic Investigation of Antimicrobial-Resistant *Salmonella enterica* Isolates from Dead Chick Embryos in China. Front. Microbiol..

[61] Wright G.D. (2007). The Antibiotic Resistome: The Nexus of Chemical and Genetic Diversity. Nat. Rev. Microbiol..

[62] Ammar A.M., Attia A.M., Abd El-Aziz N.K., Abd El Hamid M.I., El-Demerdash A.S. (2016). Class 1 Integron and Associated Gene Cassettes Mediating Multiple-Drug Resistance in Some Food Borne Pathogens. Int. Food Res. J..

[63] Castro-Vargas R.E., Herrera-Sanchez M.P., Rodriguez-Hernandez R., Rondon-Barragan I.S. (2020). Antibiotic Resistance in *Salmonella* Spp. Isolated from Poultry: A Global Overview. Vet. World.

[64] Doi Y., Iovleva A., Bonomo R.A. (2017). The Ecology of Extended-Spectrum Β-Lactamases (Esbls) in the Developed World. J. Travel. Med..

[65] Kuang D., Zhang J., Xu X., Shi W., Yang X., Su X., Shi X., Meng J. (2018). Increase in Ceftriaxone Resistance and Widespread Extended-Spectrum Β-Lactamases Genes among *Salmonella enterica* from Human and Nonhuman Sources. Foodborne Pathog. Dis..

[66] Chopra I., Roberts M. (2001). Tetracycline Antibiotics: Mode of Action, Applications, Molecular Biology, and Epidemiology of Bacterial Resistance. Microbiol. Mol. Biol. Rev..

[67] Deekshit V.K., Kumar B.K., Rai P., Srikumar S., Karunasagar I., Karunasagar I. (2012). Detection of class 1 integrons in *Salmonella* Weltevreden and silent antibiotic resistance genes in some seafood-associated nontyphoidal isolates of *Salmonella* in south-west coast of India. J. Appl. Microbiol..

[68] Neuert S., Nair S., Day M.R., Doumith M., Ashton P.M., Mellor K.C., Jenkins C., Hopkins K.L., Woodford N., de Pinna E. (2018). Prediction of Phenotypic Antimicrobial Resistance Profiles from Whole Genome Sequences of Non-typhoidal *Salmonella enterica*. Front. Microbiol..

[69] Galán-Relaño Á., Sánchez-Carvajal J.M., Gómez-Gascón L., Vera E., Huerta B., Cardoso-Toset F., Gómez-Laguna J., Astorga R.J. (2022). Phenotypic and genotypic antibiotic resistance patterns in *Salmonella* Typhimurium and its monophasic variant from pigs in southern Spain. Res. Vet. Sci..

[70] Schwan C.L., Lomonaco S., Bastos L.M., Cook P.W., Maher J., Trinetta V., Bhullar M., Phebus R.K., Gragg S., Kastner J. (2021). Genotypic and Phenotypic Characterization of Antimicrobial Resistance Profiles in Non-typhoidal *Salmonella enterica* Strains Isolated from Cambodian Informal Markets. Front. Microbiol..

[71] Liu Q., Chen W., Elbediwi M., Pan H., Wang L., Zhou C., Zhao B., Xu X., Li D., Yan X. (2020). Characterization of *Salmonella* Resistome and Plasmidome in Pork Production System in Jiangsu, China. Front. Vet. Sci..

[72] Kaye K.S., Pogue J.M., Tran T.B., Nation R.L., Li J. (2016). Agents of Last Resort: Polymyxin Resistance. Infect. Dis. Clin. N. Am..

