# Supplementary material for: Comparative Analysis between Salmonella enterica Isolated from Imported and Chinese Native Chicken Breeds
Source: Microorganisms. 2023 Feb 3;11(2):390. doi: 10.3390/microorganisms11020390 (PMC9962450; doi:10.3390/microorganisms11020390)
Supplement: Supplementary file 1 [file microorganisms-11-00390-s001.zip › microorganisms-2129146-supplementary.pdf]

Table S1. The primers used for confirming *Salmonella* and S. Pullorum.

Target gene	Primer sequence（5'-3'）	Product size（bp）	Annealing temperature (℃)	Reference
IpaJ	F TACCTGTCTGCTGCCGTGA<br>R ACCCTGCAAACCTGAAATC	740	58	[10]
FimW	F AACAGTCACTTTGAGCATGGGTT<br>R GAGTGACTTTGTCTGCTCTTCA	477	50	[38]

Table S2. The primer sequences of antimicrobial resistance gene.

Target gene	Primer sequence（5'-3'）	Annealing temperature (℃)	Reference
bla_{TEM}	F ATAAAATTCTTGAAGACGAAA<br>R GACAGTTACCAATGCTTAATC	57	[43]
bla_{SHV}	F TTATCTCCCTGTTAGCCACC<br>R GATTTGCTGATTTCGCTCGG	57	[43]
bla_{CTX-M}	F CGCTTTGCGATGTGCAG<br>R ACCGCGATATCGTTGGT	54	[44]
bla_{VIM}	F GATGGTGTTTGGTCGCATA<br>R CGAATGCGCAGCACCAG	55	[44]
bla_{NDM}	F GGTTTGGCGATCTGGTTTTC<br>R CGGAATGGCTCATCACGATC	52	[44]
bla_{IMP}	F GGAATAGAGTGGCTTAAYTCTC<br>R GGTTTAAYAAAACAACCACC	60	[44]
bla_{MIR}	F TCG GTAAAGCCGATGTTGCGG<br>R CTT CCACTGCGGCTGCCAGTT	64	[45]
bla_{DHA}	F AACTTTCACAGGTGTGCTGGGT<br>R AACTTTCACAGGTGTGCTGGGT	64	[45]
qnrC	F GGTTGTACATTTATTGAATC<br>R TCCACTTTACGAGGTTCT	50	[46]
oqxA	F GATCAGTCAGTGGGATAGTTT<br>R TACTCGGCGTTAACTGATTA	55	[47]

AAC2	F ACTGTGATGGGATACGCGTC<br>R CTCCGTCAGCGTTTCAGCTA	58	[48]
AAC4	F CTTCAGGATGGCAAGTTGGT<br>R TCATCTCGTTCTCCGCTCAT	58	[48]
tetA	F GCGCCTTTCCTTTGGGTTCT<br>R CCACCCGTTCCACGTTGTTA	62	[48]
tetB	F CATTAATAGGCGCATCGCTG<br>R TGAAGGTCATCGATAGCAGG	57	[48]
tetC	F TTGCGGGATATCGTCCATTC<br>R CATGCCAACCCGTTCCATGT	62	[48]
sul1	F CTTCGATGAGAGCCGGCGGC<br>R GCAAGGCGGAAACCCGCGCC	58	[49]
sul2	F GCGCTCAAGGCAGATGGCATT<br>R GCGTTTGATACCGGCACCCGT	50	[49]
Mcr-1	F CGGTCAGTCCGTTTGTTC<br>R CTTGGTCGGTCTGTAGGG	55	[28]
Mcr-2	F TGTTGCTTGTGCCGATTGGA<br>R AGATGGTATTGTTGGTTGCTG	65	[28]
Mcr-3	F TTGGCACTGTATTTTGCATTT<br>R TTAACGAAATTGGCTGGAACA	50	[28]
Mcr-4	F ATTGGGATAGTCGCCTTTTT<br>R TTACAGCCAGAATCATTATCA	56	[50]

Table S3. The PCR conditions and size of PCR products among 21 antimicrobial resistance genes.

**Target gene**	**Procedures**						**Amplicon size(bp)**	**Reference**
	**Pre-denaturation**	**Denaturation**	**Annealing**	**Elongation**	**Cycles**	**Elongation**		
bla_{TEM}	94 5min	94℃ 60s	57℃ 60s	72℃ 1min	35	72℃ 10min	643	[43]
bla_{SHV}	94℃ 5min	94℃ 30s	57℃ 30s	72℃ 1min	30	72℃ 10min	860	[43]
$bla_{\text{CTX-M}}$	94℃ 5min	94℃ 30s	54℃ 30s	72℃ 1min	32	72℃ 10min	550	[44]
bla_{VIM}	94℃ 10min	94℃ 30s	55℃ 40s	72℃ 45s	30	72℃ 10min	390	[44]
bla_{NDM}	94℃ 10min	94℃ 30s	52℃ 40s	72℃ 50S	36	72℃ 5min	621	[44]
bla_{IMP}	94℃ 10min	94℃ 30s	60℃ 40s	72℃ 50S	36	72℃ 5min	232	[44]
bla_{MIR}	94℃ 3min	94℃ 30s	64℃ 30s	72℃ 60S	25	72℃ 7min	302	[45]

bla$_{\text{DHA}}$	94℃ 3min	94℃ 30s	64℃ 30s	72℃ 60S	25	72℃ 7min	405	[45]
qnrC	94℃ 5min	94℃ 30s	50℃ 30s	72℃ 1min	32	72℃ 10min	666	[46]
oqxA	94℃ 5min	94℃ 30s	55℃ 30s	72℃ 1min	32	72℃ 10min	670	[47]
AAC2	95℃ 5min	94℃ 30s	58℃ 30s	72℃ 1min	32	72℃ 10min	482	[48]
AAC4	95℃ 5min	94℃ 30s	58℃ 30s	72℃ 1min	32	72℃ 10min	286	[48]
tetA	95℃ 5min	94℃ 30s	50℃ 30s	72℃ 45s	30	72℃ 10min	937	[48]
tetB	95℃ 5min	94℃ 30s	50℃ 30s	72℃ 45s	30	72℃ 10min	416	[48]
tetC	95℃ 10min	95℃ 30s	55℃ 60s	72℃ 60s	30	72℃ 7min	510	[48]
sul1	95℃ 5min	94℃ 30s	58℃ 30s	72℃ 45s	28	72℃ 10min	238	[49]
sul2	95℃ 5min	94℃ 30s	50℃ 30s	72℃ 45s	32	72℃ 10min	793	[49]
Mcr-1	94℃ 4min	94℃ 30s	55℃ 60s	72℃ 60s	30	72℃ 10min	309	[28]
Mcr-2	95℃ 3min	95℃ 30s	55℃ 30s	72℃ 60s	30	72℃ 10min	567	[28]
Mcr-3	94℃ 4min	94℃ 30s	50℃ 60s	72℃ 60s	30	72℃ 10min	542	[28]
Mcr-4	94℃ 4min	94℃ 30s	56℃ 60s	72℃ 60s	32	72℃ 10min	487	[50]

Table S4: The characteristics of 94 *Salmonella* isolates.

**Isolate**	**Location**	**Farm**	**breed**	**Sreotype**	**ESBL phenotype**	**Resistance profiles**	**Resistance genes**
20TA01	Tai'an	Farm A	White feather broiler	*S.* Enteritidis	-	TET-DOX-STR-AMP	*bla*$_{\text{TEM}}$, *tetA*, *tetB*, *sul1*
20TA02	Tai'an	Farm A	White feather broiler	*S.* Enteritidis	-	TET-DOX-STR-AMX-AMP	*bla*$_{\text{TEM}}$, *tetA*, *sul1*
20TA03	Tai'an	Farm A	White feather broiler	*S.* Enteritidis	-	TET-DOX-STR-AMX-AMP	*bla*$_{\text{TEM}}$, *tetA*, *tetB*, *sul1*
20TA04	Tai'an	Farm A	White feather broiler	*S.* Enteritidis	-	TET-DOX-STR-AMP	*bla*$_{\text{TEM}}$, *tetA*, *tetB*, *sul1*
20TA05	Tai'an	Farm A	White feather broiler	*S.* Enteritidis	-	TET-DOX-STR-AMX-AMP	*bla*$_{\text{TEM}}$, *sul1*
20TA06	Tai'an	Farm A	White feather broiler	*S.* Enteritidis	-	TET-DOX-STR-AMX-AMP	*bla*$_{\text{TEM}}$, *tetA*, *tetB*, *sul1*

20TA07	Tai'an	Farm A	White feather broiler	*S.* Enteritidis	-	TET-DOX-STR-AMX-AMP	*bla*$_{TEM}$, *tetA*
20TA08	Tai'an	Farm A	White feather broiler	*S.* Enteritidis	-	TET-DOX-STR-AMX-AMP	*bla*$_{TEM}$
20TA09	Tai'an	Farm A	White feather broiler	*S.* Enteritidis	-	TET-STR-AMX-AMP	*bla*$_{TEM}$, *tetA*
20TA10	Tai'an	Farm A	White feather broiler	*S.* Enteritidis	-	TET-DOX-STR-AMX-AMP	*bla*$_{TEM}$, *tetA*, *tetB*, tetC, *sul1*
20TA11	Tai'an	Farm A	White feather broiler	*S.* Enteritidis	-	TET-DOX-GM-STR-AMX-AMP	*bla*$_{TEM}$, *AAC2*, *tetA*, *tetB*, *sul1*
20TA12	Tai'an	Farm A	White feather broiler	*S.* Enteritidis	-	TET-DOX-STR-AMX-AMP	*bla*$_{TEM}$, *tetA*, *sul1*
20TA13	Tai'an	Farm A	White feather broiler	*S.* Enteritidis	-	TET-DOX-STR-AMX-AMP	*bla*$_{TEM}$, *tetA*, *tetB*
20TA14	Tai'an	Farm A	White feather broiler	*S.* Enteritidis	-	TET-DOX-STR-AMX-AMP	*bla*$_{TEM}$
20TA15	Tai'an	Farm A	White feather broiler	*S.* Enteritidis	-	STR-AMX-AMP	*bla*$_{TEM}$, *tetA*, *tetB*
20TA16	Tai'an	Farm A	White feather broiler	*S.* Enteritidis	-	TET-DOX-STR-AMX-AMP	*bla*$_{TEM}$, *tetA*, *tetB*, *sul1*
20TA17	Tai'an	Farm A	White feather broiler	*S.* Enteritidis	-	TET-STR-AMP	*bla*$_{TEM}$, *tetA*, *tetB*
20TA18	Tai'an	Farm A	White feather broiler	*S.* Enteritidis	-	AMX-AMP-FOX	*bla*$_{TEM}$, *tetA*, *tetB*, *sul1*

20TA19	Tai'an	Farm A	White feather broiler	*S.* Enteritidis	-	AMX-AMP	*bla*TEM, *tetA*, *tetB*, *sul1*
20TA20	Tai'an	Farm A	White feather broiler	*S.* Enteritidis	-	STR-AMX-AMP	*bla*TEM, *tetA*, *tetB*, *sul1*
20TA21	Tai'an	Farm A	White feather broiler	*S.* Enteritidis	-	TET-AMX-AMP	*bla*TEM, *tetA*, *tetB*, *sul1*
20TA22	Tai'an	Farm A	White feather broiler	*S.* Enteritidis	-	TET-AMX-AMP	*bla*TEM, *tetA*, *tetB*, *sul1*
20TA23	Tai'an	Farm A	White feather broiler	*S.* Enteritidis	-	TET-DOX-STR-AMX-AMP	*bla*TEM, *tetA*, *tetB*, *sul1*
20JN01	Jining	Farm B	Bairi	*S.* Pullorum	-	STR-AMX-AMP	*bla*TEM, *tetA*
20JN02	Jining	Farm B	Bairi	*S.* Pullorum	-		*bla*TEM
20JN03	Jining	Farm B	Bairi	*S.* Pullorum	-	AMX-AMP	*bla*TEM, *tetA*, *tetB*
20JN04	Jining	Farm B	Bairi	*S.* Enteritidis	-	TET-DOX-STR-AMX-AMP	*bla*TEM, *tetB*, *sul1*
20JN05	Jining	Farm B	Bairi	*S.* Pullorum	-	AMX-AMP	*bla*TEM, *tetB*
20JN06	Jining	Farm B	Bairi	*S.* Pullorum	-	STR-AMX-AMP	*bla*TEM, *tetA*, *tetB*
20JN07	Jining	Farm B	Bairi	*S.* Pullorum	-	AMX-AMP	*bla*TEM, *tetA*
20JN08	Jining	Farm B	Bairi	*S.* Enteritidis	-	TET-DOX-STR-AMX-AMP	*bla*TEM, *tetA*
20JN09	Jining	Farm B	Bairi	*S.* Pullorum	-	AMP	*bla*TEM, *tetA*, *tetB*, *sul1*
20JN10	Jining	Farm B	Bairi	*S.* Pullorum	-	STR-AMX-AMP	*bla*TEM, *tetA*, *tetB*, *sul1*

20JN11	Jining	Farm B	Bairi	*S.* Pullorum	-	STR-AMX-AMP	*bla*TEM, *bla*CTX-M, *tetA*, *tetB*, *sul1*
20JN12	Jining	Farm B	Bairi	*S.* Pullorum	-	AMX-AMP	*bla*TEM, *tetA*, *tetB*, *sul1*
20JN13	Jining	Farm B	Bairi	*S.* Pullorum	-		*bla*TEM, *tetA*, *tetB*, *sul1*
20JN14	Jining	Farm B	Bairi	*S.* Enteritidis	-	TET-DOX-STR-AMX-AMP	*bla*TEM, *tetA*, *tetB*, *sul1*
20JN15	Jining	Farm B	Bairi	*S.* Pullorum	-	TET-DOX-STR-AMX-AMP	*bla*TEM, *tetA*, *tetB*, *sul1*
20SD01	Rizhao	Farm C	Langya	*S.* Thompson	-	SXT-GM-STR-AMX-AMP	*bla*TEM
20SD02	Rizhao	Farm C	Langya	*S.* Thompson	-	SXT-GM-STR-AMX-AMP	*bla*TEM
20SD03	Rizhao	Farm C	Langya	*S.* Thompson	-	SXT-GM-STR-AMX-AMP	*bla*TEM
20SD04	Rizhao	Farm C	Langya	*S.* Thompson	-	SXT-GM-STR-AMX-AMP	*bla*TEM
20SD05	Rizhao	Farm C	Langya	*S.* Thompson	-	SXT-GM-STR-AMX-AMP	*bla*TEM
20SD06	Rizhao	Farm C	Langya	*S.* Thompson	-	SXT-GM-STR-AMX-AMP	*bla*TEM
20SD07	Rizhao	Farm C	Langya	*S.* Thompson	-	SXT-GM-STR-	*bla*TEM

						AMX-AMP	
20SD08	Rizhao	Farm C	Langya	*S.* Thompson	-	SXT-TET-DOX-GM-STR-AMX-AMP	*bla*$_{TEM}$
20SD09	Rizhao	Farm C	Langya	*S.* Thompson	-	SXT-GM-STR-AMX-AMP	*bla*$_{TEM}$
20SD10	Rizhao	Farm C	Langya	*S.* Thompson	-	SXT-TET-DOX-GM-STR-AMX-AMP	*bla*$_{TEM}$
20SD11	Rizhao	Farm C	Langya	*S.* Thompson	-	SXT-GM-STR-AMX	*bla*$_{TEM}$
20SD12	Rizhao	Farm C	Langya	*S.* Thompson	-	SXT-GM-STR-AMX-AMP	*bla*$_{TEM}$, *tetA*
20SD13	Rizhao	Farm C	Langya	*S.* Thompson	-	SXT-GM-STR-AMX-AMP	*bla*$_{TEM}$, *tetB*
20SD14	Rizhao	Farm C	Langya	*S.* Thompson	-	SXT-GM-STR-AMX	*bla*$_{TEM}$
20SD15	Rizhao	Farm C	Langya	*S.* Thompson	-	SXT-GM-STR-AMX-AMP	*bla*$_{TEM}$, *sul2*
20SD16	Rizhao	Farm C	Langya	*S.* Thompson	-	SXT-GM-STR-AMX-AMP	*bla*$_{TEM}$
20SD17	Rizhao	Farm C	Langya	*S.* Thompson	-	SXT-GM-AMX-AMP	*bla*$_{TEM}$
20SD18	Rizhao	Farm C	Langya	*S.* Thompson	-	GM-STR-AMX-AMP	*bla*$_{TEM}$
20SD19	Rizhao	Farm C	Langya	*S.* Thompson	-	SXT-GM-STR-	*bla*$_{TEM}$

						AMX-AMP	
20SD20	Rizhao	Farm C	Langya	*S.* Thompson	-	SXT-GM-STR-AMX-AMP	*bla*$_{TEM}$
20SD21	Rizhao	Farm C	Langya	*S.* Thompson	-	SXT-GM-STR-AMX-AMP	*bla*$_{TEM}$
20SD22	Rizhao	Farm C	Langya	*S.* Thompson	-	SXT-GM-STR-AMX-AMP	*bla*$_{TEM}$
20SD23	Rizhao	Farm C	Langya	*S.* Thompson	-	SXT-GM-STR-AMX-AMP	*bla*$_{TEM}$
20SD24	Rizhao	Farm C	Langya	*S.* Thompson	-	SXT-GM-STR-AMX-AMP	*bla*$_{TEM}$, *tetB*
20SD25	Rizhao	Farm C	Langya	*S.* Thompson	-	SXT-GM-STR-AMX-AMP	blaTEM
20SD26	Rizhao	Farm C	Langya	*S.* Thompson	-	SXT-GM-STR-AMX-AMP	*bla*$_{TEM}$, *tetA*, *tetB*
20SD27	Rizhao	Farm C	Langya	*S.* Thompson	-	SXT-GM-STR-AMX-AMP	*bla*$_{TEM}$
20SD28	Rizhao	Farm C	Langya	*S.* Thompson	-	SXT-GM-STR-AMX-AMP	*bla*$_{TEM}$
20SD29	Rizhao	Farm C	Langya	*S.* Thompson	-	SXT-GM-AMX-AMP	*bla*$_{TEM}$
20SD30	Rizhao	Farm C	Langya	*S.* Thompson	-	SXT-AMX-AMP	*bla*$_{TEM}$

20SD31	Rizhao	Farm C	Langya	*S.* Thompson	-	STR-AMX-AMP	*bla*TEM, *tetA*
20SD32	Rizhao	Farm C	Langya	*S.* Thompson	-	STR-AMX-AMP	*bla*TEM
20LH06	Jining	Farm D	Luhua	*S.* Pullorum	+	STR-CAZ	*bla*TEM, *tetB*
20LH07	Jining	Farm D	Luhua	*S.* Pullorum	+	STR-CAZ	*bla*TEM
20LH08	Jining	Farm D	Luhua	*S.* Pullorum	-	STR-CAZ	*bla*TEM, *tetB*
20LH09	Jining	Farm D	Luhua	*S.* Pullorum	-	STR-CAZ	*bla*TEM
20LH10	Jining	Farm D	Luhua	*S.* Pullorum	-	STR-CAZ	*bla*TEM, *tetA*, *tetB*
20LH11	Jining	Farm D	Luhua	*S.* Pullorum	+	STR-CAZ	*bla*TEM, *tetA*, *tetB*
20LH12	Jining	Farm D	Luhua	*S.* Pullorum	+	TET-STR-AMX-AMP-CAZ	*bla*TEM, *tetB*
20LH13	Jining	Farm D	Luhua	*S.* Pullorum	-	STR-CAZ	*bla*TEM
20LH14	Jining	Farm D	Luhua	*S.* Pullorum	-	TET-STR	*bla*TEM, *tetA*
20LH15	Jining	Farm D	Luhua	*S.* Pullorum	-	STR-FOX-CAZ	*bla*TEM
20LH16	Jining	Farm D	Luhua	*S.* Pullorum	+	STR-AMX-AMP-CAZ	*bla*TEM, *tetB*
20LH17	Jining	Farm D	Luhua	*S.* Pullorum	+	STR-CAZ	*bla*TEM, *tetA*, *tetB*, *sul1*, *sul2*
20LH18	Jining	Farm D	Luhua	*S.* Pullorum	-	STR-AMX-AMP-CAZ	*bla*TEM, *tetB*
20LH19	Jining	Farm D	Luhua	*S.* Pullorum	-	AMX-AMP	*bla*TEM, *tetA*, *tetB*, *sul1*
20LH20	Jining	Farm D	Luhua	*S.* Pullorum	-	STR-CAZ	*bla*TEM, *tetA*, *tetB*

20LH21	Jining	Farm D	Luhua	*S.* Pullorum	-	STR-CAZ	*bla*$_{TEM}$
20LH22	Jining	Farm D	Luhua	*S.* Pullorum	+	STR-CAZ	*bla*$_{TEM}$, *tetA*, *tetB*
20LH23	Jining	Farm D	Luhua	*S.* Pullorum	-	STR-CAZ	*bla*$_{TEM}$, *tetA*, *tetB*
20LH24	Jining	Farm D	Luhua	*S.* Pullorum	-	STR-AMX-AMP-FOX	*bla*$_{TEM}$, *tetA*, *tetB*
20LH25	Jining	Farm D	Luhua	*S.* Pullorum	-	STR-CAZ	*bla*$_{TEM}$
20LH26	Jining	Farm D	Luhua	*S.* Pullorum	+	CAZ	*bla*$_{TEM}$
20LH27	Jining	Farm D	Luhua	*S.* Pullorum	-	TET-STR-CAZ	*bla*$_{TEM}$
20LH28	Jining	Farm D	Luhua	*S.* Pullorum	-	STR	*bla*$_{TEM}$
20LH29	Jining	Farm D	Luhua	*S.* Pullorum	-	STR	*bla*$_{TEM}$
